# A naturally occurring NS1 variant with effector domain deletion gains growth advantages in influenza virus infection

**DOI:** 10.1080/22221751.2025.2556731

**Published:** 2025-09-03

**Authors:** Ruei-Sheng Tsai, Jun-Yuh Tsai, Yun-Ting Chung, Ching-Yu Tseng, Guan-Ru Liao, Randy A. Albrecht, Chih-Ying Kuan, Rei-Lin Kuo, Shan-Chia Ou, Wei-Li Hsu

**Affiliations:** a Graduate Institute of Microbiology and Public Health, College of Veterinary Medicine, National Chung Hsing University, Taichung, Taiwan; b Department of Life Science, College of Life Science, National Chung Hsing University, Taichung, Taiwan; c Department of Microbiology, Global Health and Emerging Pathogens Institute, Icahn School of Medicine at Mount Sinai, New York, NY, United States; d The iEGG and Animal Biotechnology Center, National Chung Hsing University, Taichung, Taiwan; e Doctoral Program in Microbial Genomics, National Chung Hsing University and Academia Sinica, Taichung, Taiwan; f Research Center for Emerging Viral Infections, College of Medicine, Chang Gung University, Taoyuan, Taiwan; g Department of Medical Biotechnology and Laboratory Science, College of Medicine, Chang Gung University, Taoyuan, Taiwan

**Keywords:** Avian, influenza virus, H5N2, non-structural protein 1 (NS1), deletion, RNA binding, dimer formation, splicing

## Abstract

High pathogenicity avian influenza virus (HPAIV) poses major threats to both poultry health and public safety. The viral nonstructural protein 1 (NS1) plays a crucial role in counteracting innate immunity. NS1 typically consists of approximately 230 amino acids with two domains: an RNA-binding domain (RBD) and the effector domain (ED). A novel naturally occurring NS1 variant, termed NS100, was reported herein, isolated during propagation of a recombinant H5N2 virus. NS100 encodes only the 1–95 residues of NS1, lacking the entire ED. Surprisingly, the NS100 virus replicated as efficiently as the wild-type (WT) virus in both cell and animal models. Cells infected with NS100 exhibited reduced interferon and cytokine expression, indicating preserved immune evasion capacity. Biochemical analyses revealed that NS100 retains key NS1 functions mediated by the RBD, including dsRNA binding, PKR interaction, and homodimer formation. In addition, NS100 cooperated with the viral RNA polymerase complex in a mini-genome assay, indicating functional synergy. Interestingly, the NS100 virus showed markedly reduced NS2 protein expression, likely due to impaired splicing caused by a GA transversion at a polypyrimidine tract. Transcriptome analysis further confirmed reduced splicing efficiency of NS transcripts and lower expression of antiviral host genes in NS100-infected cells. In conclusion, our findings demonstrate that NS1 can maintain essential pro-viral functions despite the loss of its ED, and that NS100 provides new insights into the modularity of NS1 and its broader role in regulating host-virus interactions.

## Introduction

Influenza, caused by influenza A viruses (IAVs), is one of the most important respiratory diseases threatening global public health. Despite IAV infection in humans, the broad host range of the virus also affects animal wellness and impacts poultry significantly [1]. It has been reported that several subtypes of avian influenza viruses, including H5N1, H7N9 and H5N2, could infect mammalian hosts via zoonotic transmission, which promotes the virus to form novel lineage, adapt new hosts, and cause global pandemics [2, 3]. The first documented outbreak of human infection with highly pathogenic avian influenza virus (HPAIV), H5N1, which occurred in Hong Kong in 1997, devastated the poultry industries and posed a threat to human wellness [4,5]. While HPAIVs often lead to severe symptoms in hosts, infections of low pathogenic AIVs (LPAIVs) are primarily asymptomatic. In 2003, Taiwan reported its first case of LPAIV H5N2, which has since become endemic in chickens. Co-circulation with endemic H6N1 led to H5N2 evolving into HPAIV in 2012, causing severe poultry outbreaks [6,7]. Phylogenetic analyses indicate that the HA and NA segments of this H5N2 strain, known as Mexican-like H5N2, originated from Mexico, while the remaining six genes came from local H6N1 viruses[7]. This highlights the virus's adaptation through multiple gene reassortments. Moreover, host adaptations drive viral mutations in viral proteins that facilitate binding to host cell receptors or enhance viral replication efficiency in the new host.

During IAV infection, the presence of dsRNA triggers interferon (IFN) responses and downstream signalling, including PKR phosphorylation, which activates the α subunit of eukaryotic initiation factor-2 (eIF-2α), thereby dampening the translation of viral and cellular proteins [8]. Viral nonstructural protein 1 (NS1), a multifunctional protein, plays a significant role in counteracting innate immunity. NS1, spanning 230 to 237 amino acids, features two core functional domains connected by the linker region [9]. The N-terminal RNA-binding domain (RBD, residues 1-73) binds dsRNA and counteracts IFN α/β responses. Additionally, the C-terminal effector domain (ED, residues 88-202) acts as an interface for interacting with and compromising the function of cellular partners[10,11]. For instance, through the ED, NS1 associates with cytoplasmic antiviral proteins such as PKR and CPSF30, which lead to suppressing IFN and pro-inflammatory cytokine production, ultimately curtails host antiviral responses and mRNA maturation, respectively[12,13]. Notably, the formation of a homodimer via both RBD and ED interphase is a critical structural alteration for NS1 to interact with dsRNA or cellular counterparts, representing a pivotal mechanism for counteracting host immune responses [14,15].

To gain more advantages in combating host defense, IAVs are frequently mutating throughout the dynamic evolution. As a critical virulence factor during infection, NS1 is pivotal in this process, undergoing genetic changes and recombination, leading to new pathogenic strains [16]. NS1 mutations often dampen IFN responses [17,18], while the D92Q mutation in HPAIV H5N1 NS1 enhances virulence and cytokine resistance [19,20]. However, NS1-truncated viruses typically exhibit reduced virulence and impaired replication [21–23]. Recently, our study uncovered another strategy: alongside the full-length NS1 protein, HPAIV H5N2 (strain NS1031) encodes an additional NS1 variant, NS3, resulting from a single nucleotide substitution that introduces a novel splicing donor site on the NS1 RNA [24]. NS3 is a novel truncated variant (missing residue 126-168) of the NS1 protein generated through alternative splicing of the NS1 gene. Unlike the full-length NS1(T375G), which facilitates viral growth in avian cells, H5N2 NS3 enhances viral replication and pathogenicity in mammalian systems by interacting with and suppressing the activation of PKR, the dsRNA-dependent serine/threonine protein kinase R, despite lacking a significant portion of the NS1 protein. Notably, NS3 has been identified not only in avian H5N2 but also in the H3N2 subtype during serial passages of this human-origin virus in mice [25]. This process led to specific mutations in the H3N2 NS1 sequence, thereby yielding the NS3 spliced RNA, which in turn facilitates systemic infection and broadens tissue tropism. Both studies highlight the critical role of NS3 in the adaptation and increased virulence of influenza viruses in new host species.

A variant influenza virus, designated as NS100, was isolated during the generation of a reassortant virus expressing the full-length NS1 derived from H5N2 (strain NS1031), known as RG-AIV-T375G [24]. While deletion of NS1 typically leads to viral attenuation, the successful rescue of NS100 suggests that the partial deletion of ED in this virus is not detrimental. In this study, we characterized the NS100 virus and investigated its mechanism of growth advantage. These findings demonstrate that NS100 preserves the biological functions of NS1 and may inhibit host immune responses through multiple mechanisms.

## Material and methods

### Cells and viruses

Canine MDCK cells, chicken DF-1 cells, human A549 cells, and human embryonic kidney HEK293T cells were maintained in Dulbecco’s modified Eagle’s medium (DMEM, Gibco BRL, Life Technologies Corporation Carlsbad, CA, USA), while mouse M1 cells were culture in DMEM-F1, supplemented with 10% fetal bovine serum (FBS, Hyclone, Logan, UT, USA) and antibiotics.

RG-1031WT, the virus that expresses the wild-type NS1031 gene was generated in the previous study [24], while the NS100 virus, which contains a C-terminally truncated NS1 protein, was originally isolated during propagation of the RG-T375G virus that expressed only the full-length NS1 without NS3 variant [24]. The sequence of NS1031, NS100 and NS95 viruses has been deposited in GenBank with accession number: PX048677-PX048684, PX226327-PX226334 and PX289092-PX289099, respectively.

Influenza viruses were propagated in 10-day-old embryonated specific-pathogen-free (SPF) chicken eggs for 48 hr at 37°C, following a standard protocol [26].

### Generation of reassortant RG-AIV-NS95 by reverse genetics

The reassortant viruses expressing C-truncated NS95 variants, derived from the NS segment (accession number KU646889.1) of the A/goose/Taiwan/01031/2015 avian influenza virus (abbreviated as strain NS1031), were generated using reverse genetics [27]. Two segments containing untranslated regions (UTRs) and partial coding sequences for residues 1–95 were first amplified from the RG-1031WT-NS plasmid using two sets of primers. These amplicons then served as templates for overlap extension PCR to generate the full NS95 segment. Primer sequences are provided in Table 1. The resulting PCR product was subcloned into the reverse genetic vector pDZ, kindly provided by Professor Peter Palese (Icahn School of Medicine at Mount Sinai, USA) [28]. Cloning and virus generation followed the protocol described in our previous report [27]. The authenticity of the influenza virus genomes, including all segments was confirmed through NGS (Illumina) sequencing, ensuring that the observed effects are due to mutations in the NS segment. The sequence of NS95 virus has been deposited in GenBank with Submission ID: 2990926.

**Table 1.** Primer used for generation of constructs expressing NS1 variant mRNA or proteins.

Primer	Sequence (5’−3’)	Plasmids
NS1-EcoR I-F	5'-AAGAATTCATGGATTCCAACACTGTGTCAAG-3'	NS95-FLAG
NS95-Kpn I-R	5'-TTGGTACCTTAAGAGTCATATCTGTTAGGTA-3'	
NS100-BamH I-F	5'-TTTGGATCCATGGATTCCAACACTGTGTCAAG-3'	NS100-eGFP
NS100-Xho I-R	5'-TTCTCGAGTAAGCATGTCCTGGAATCAAGAG-3'	NS100-FLAG
NSWT-F2-mut-F NSWT-F1-mut-R	TTACCTTCGATTCCAGGACATGCTG CATGTCCTGGAATCGAAGGTAATGG	NS (WT)−495/496 mutation
NS100-F2-mut-F NS100-F1-mut-R	TGACTCTTTCTTCCAGGACATGCTG AGCATGTCCTGGAAGAAAGAGTCATATCTG	NS100-495/496 mutation
NS-BsmB I-F NS-BsmB I -R	TATTCGTCTCAGGGAGCAAAAGCAGGGTG ATATCGTCTCGTATTAGTAGAAACAAGGGTGTTTT	1. NS95 plasmid for reverse genetics 2. Outer primer set for NS amplification
NS1-F1-Bgl II	5'-AAAGATCTATGGATTCCAACACTGTGTCAAG-3'	NS95-eGFP
NS95-Not I-eGFP-R	5'-TTGCGGCCGCAAGAGTCATATCTGTTAGGTA-3'	

The underlined nucleotides indicated the PY-tract mutation site.

### Generation of constructs expressing NS100 and NS95

Two sets of constructs were generated for the expression of NS100 or NS95 proteins with the fusion of either a 3xFLAG-tag or an eGFP protein at their C-terminus. PCR was used to amplify the coding region of the two NS1 variants. Primers are available in Table 1. Wild-type NS1031-FLAG plasmid [27] served as the template for the amplification of the NS100 coding region by PCR, and subsequently, the resulting NS100-FLAG plasmid was used for NS95 amplification.

After PCR amplification, the resulting amplicon bearing the NS100 fragment was subsequently treated with restriction enzymes *Bam*H I and *Xho* I to clone into vector pcDNA6, which contains a flanking C-terminal 3xFLAG-tag or eGFP, respectively. While, the PCR product for NS95 was treated with two sets of restriction enzyme, *Eco*R I/*Kpn* I or *Bgl* II/*Not* I, for cloning into vector pCMV14 (linearized with *Eco*R I/*Kpn* I) or GFP-pcDNA3.1 (linearized with *Bam*H I/Not I), which expresses an FLAG-tag and eGFP fusion protein, respectively.

### Plaque assay

MDCK cells, seeded in 12-well plates (approximately 90% confluency), were washed once with PBS and infected with 400 μL/well of serially diluted viruses in the infectious medium (DMEM with TPCK-treated trypsin at 1.0 µg/mL). After 3 hours of incubation, the cells were washed with PBS and overlaid with the infectious medium containing 0.6% agarose. At 48 hours post-infection (hpi), cells were fixed with 10% formaldehyde, and plaques were visualized using crystal violet staining. The virus titre was determined and expressed as the mean plaque-forming units (PFU) per mL.

### Growth kinetics of reassortant AIVs

MDCK, A549, M1, and DF-1cells were infected with the reassortant virus expressing either wild-type NS1 (RG-AIV-WT) or NS100 viruses at a multiplicity of infection (MOI) of 0.01. The culture medium containing viruses was collected at 12, 24, 36, and 48 hpi. The titres of viral progenies in the culture medium were determined by plaque assay. The results were shown as relative titres with RG-AIV-WT at each time point.

### Transfection

Transfection was performed using Lipofectamine 2000 (Invitrogen, Carlsbad, CA, USA) following the manufacturer’s instructions. Briefly, cells were seeded in a 12-well plate the night before transfection to achieve approximately 70%∼80% confluency. A total of 2 μg of plasmid DNA was mixed with 4 μL of Lipofectamine, diluted in 100 μL of DMEM (without FBS or antibiotics), and incubated at room temperature for 20 minutes. The DNA-liposome mixture was then added dropwise to the cell monolayers and incubated for 24 hours before further analysis.

### Western blot analysis

Whole-cell lysates were prepared and separated by sodium dodecyl sulfate-polyacrylamide gel electrophoresis (SDS-PAGE) followed by electro-transfer onto nitrocellulose (NC) paper (Bio-Rad). The filter was blocked in 5% skim milk for 1 h and then incubated with the primary antibodies at 4°C for overnight. Subsequently, the filter was rinsed with PBS containing 0.05% Tween 20 (PBS-T) for five times, followed by incubation with the corresponding secondary antibody conjugated with horseradish peroxidase (HRP) for 1 h. After washing with PBS-T, protein signals were detected by enhanced chemiluminescence (ECL) and acquired by ImageQuant LAS 4000 (GE Healthcare, Uppsala, Sweden). The dilutions of each antibody were as follows: anti-FLAG (1:2,500; Signalway Antibody), anti-β-actin (1:5,000; Jackson), anti-NS1 (1:2,000, GeneTex), anti-NS2 (1:5000, Invitrogen, USA), anti-NP (1:1,000; GeneTex), anti-PKR (1:2,000, Abcam), anti-phosphorylated PKR (1:2,000, Abcam), anti-eIF-2α (1:2000, Cell Signalling Technology), and anti-phosphorylated eIF-2α (1:1000, Abcam). Ultimately, the signal was quantified by using Image-J software.

### Poly(I:C) pull-down assay

Poly(I:C) pull-down assays were performed as previously described [29,30]. Briefly, poly(C)-coated agarose (Sigma-Aldrich) was mixed with two volumes of 2 mg/ml poly(I) (Sigma-Aldrich) in Tris-buffered saline (TBS) [50 mM Tris, pH 7.5; 150 mM NaCl] and incubated overnight at 4°C with gentle rocking. The poly(I:C) agarose beads were harvested, washed with TBS, and resuspended in binding buffer A [50 mM Tris, pH 7.5; 150 mM NaCl; 1 mM EDTA; 1% Nonidet P-40]. To assess the dsRNA binding ability of NS1 variants, poly(I:C) beads were mixed with cell lysates containing NS1 variants and incubated at 4°C for 1 hour. The poly(I:C) beads were collected by centrifugation at 800 × g and washed twice with buffer C. (10 mM Tris-HCl [pH 7.5], 6 mM MgCl_2_, 80 mM KCl, 2 mM dithiothreitol, 250 mM sucrose, and 0.1 mM EDTA) and were resuspended in SDS sample dye.

### RNA sequencing

Human A549 cell were infected with either RG-AIV-WT, NS100 or left untreated (Mock) at an MOI of 0.5. At the 12 hpi, total RNA was extracted from infected cells by TRIzol® method (Invitrogen, USA). The construction of library was generated following the TruSeq Stranded mRNA Library Prep Kit (Illumina, San Diego, CA, USA) by Genomics BioSci & Tech. Co.Ltd, (New Taipei city, Taiwan). The cDNA library was then sequenced for paired-end, 150-bp, sequenced on NovaSeq 6000 system (Illumina, USA). The experiment was conducted in three independent repeats.

### Differential gene expression (DGE) analysis

Based on the RNA sequencing data, comparative transcriptome analysis was carried out in various groups: (1) WT vs. Mock (2) NS100 vs. Mock (3) NS100 vs. Mock. Each raw reads of samples were filtered to remove adapter sequences and low-quality reads using Trimmomatic [31]. The filtered reads were aligned to the human h19 transcript sequences using Bowtie2 [32]. The aligned reads were counted by RSEM [33] then input into DEseq2 (version 1.34.0) [34] in R version 4.1.3 to generate the differential gene expression results. The cutoff of adjusted *p*-value < 0.01 with fold change ≥ 2 or ≤ −2 was used to identify genes with significant expression changes. EnhancedVolcano (version 1.12.0) was used to visualize DGE results.

Twenty-seven genes related to Type I/III interferon and ISGs were selected for expression profiling. The read counts of selected genes were transformed to Z-scores for each gene, which were then used to generate a heatmap with hierarchical clustering using R package pheatmap (version 1.0.12) based on Euclidean distance. The Log2 TPM values of selected genes were transformed to Z-scores for each gene, which were then used to generate a heatmap with hierarchical clustering using R package pheatmap (version 1.0.12) based on Euclidean distance.

### Functional enrichment analysis

To evaluate the significance of the biological functions of down-regulated genes in the NS100 compared with the mock group, we used the Database for Annotation, Visualization, and Integrated Discovery (DAVID; version 2021) [35]. The biological processes of Gene Ontology (GO) terms were analyzed to identify enriched GO terms. The top 20 enriched terms were visualized using R package ggplot2 (version 3.4.1).

### Construction of plasmids expressing NS viral RNA with mutations at the polypyrimidine tract

Constructs expressing negative-sense NS viral RNA were generated to mimic virus RNA transcription. The NS segment with double-mutation introduced at residues 495 and 496, which are located at the predicted polypyrimidine tract near the NS2 acceptor site, was generated by overlap extension PCR using the specific primer sets, as listed in Table 1. The resulting PCR products containing NS sequences were digested with *Bsm*B I and subsequently cloned into the pPoL I plasmid, driven by human polymerase I promoter, which was modified from the pHW2000 plasmid [36]. The identity of the plasmid was validated by automated sequencing (Mission Biotech Inc., Taipei, Taiwan).

### The mini-genome reporter assay for evaluation of viral replication and splicing efficiency

The viral RNA polymerase activity was evaluated by the mini-genome reporter assay adapted from a previously published method [24]. Initially, human 293 T cells were plated in 48-well plates and co-transfected with plasmids encoding different NS1 variants (100 ng each), components of the H1N1 (PR8 strain) RdRp complex – NP, PB1, PB2, and PA (20 ng each) – a firefly luciferase plasmid (10 ng) for normalization of transfection efficiency, and 100 ng of the reporter plasmid pPol I-Flu-Rluc. This reporter construct includes a mini-genome encoding Renilla luciferase, with transcription initiated by the human RNA polymerase I promoter and subsequent expression regulated by the influenza polymerase complex. After 24 hours, cell lysates were analyzed using the Dual-Glo Luciferase Assay System (Promega) as per the manufacturer’s instructions. Luminescence readings were obtained using a FLUOstar OPTIMA plate reader (BMG Labtech, Germany). RdRp activity was determined by calculating the ratio of Renilla to firefly luciferase signals (RLU), and results were expressed as a percentage relative to the highest RLU value in each experimental set.

Additionally, splicing profiles of NS transcript were evaluated by using the pPoL I plasmid expressing NS variant vRNA bearing double-point mutation at polypyrimidine tract were co-transfected into HEK293 T cells using Lipofectamine2000 reagent (Invitrogen, USA). After 15 h, total RNA was extracted from transfected cells by the TRIzol® method (Invitrogen, USA). To remove residual plasmid DNA, total RNA was treated with DNase I (New England Biolabs, Beverly, MA, United States) followed by extraction of Acid-Phenol: Chloroform (Invitrogen, USA). The DNase-treated total RNA was reverse-transcribed by Magic RT cDNA synthesis kit (Bio-Genesis®, Taipei, Taiwan). The splicing efficiency of the NS transcript was evaluated by the detection of NS transcripts. First, all the transcripts derived from NS segments, including unspliced NS1 and NS100, as well as spliced transcripts, i.e. NS3 and NS2, were universally amplified by RT–PCR using primers (Forward: 5′-TCCAACACTGTGTCAAGCTTTCAG, reverse: 5′-AGAATGTTCTCATCTCTTGCTCCAC) targeting the conserved sequences located at 5′ and 3′ ends of the NS segment.

### Measurement of splicing efficiency based on RNA-seq

The splicing efficiency (SE) of the NS segment in infected cells was measured by the RNA-seq approach. Briefly, the filter reads of RG-AIV-WT and NS100 virus-infected groups were aligned to each viral genome sequence using HISAT2 [37], converting and sorting the BAM file using SAMtools [38]. Then the SE was estimated based on a weighted SE method [39]. To unambiguously estimate the SE of multi-transcripts, a modified formula was used to calculate the SE of NS2 and NS3 transcripts in the WT group. The NS2 or NS3 exon-exon spliced reads (), exon-intron unspliced reads ($\mathrm{NS}2_{\mathrm{EI}}\;\mathrm{and}\;\mathrm{NS}3_{\mathrm{EI}}$) from either the NS2 or NS3 donor site region, and intron-exon unspl$\mathrm{NS}2_{\;\mathrm{jun}}\;\mathrm{and}\;\mathrm{NS}3_{\;\mathrm{jun}}$iced reads($\mathrm{Commo}n_{\mathrm{IE}}$) from the common NS2/NS3 acceptor site were extracted using SAMtools. The weighted index *P* were calculated as follows:

(1) $$\;P{\;}_{\mathrm{NS}2\;}=\frac{\sum \mathrm{NS}2_{\mathrm{EI}}\;}{\sum \mathrm{NS}2_{\mathrm{EI}}+\sum \mathrm{NS}3_{\mathrm{EI}}+\sum \mathrm{Commo}n_{\mathrm{IE}}}$$

(2) $$\;P{\;}_{\mathrm{NS}3}=\frac{\sum \mathrm{NS}3_{\mathrm{EI}}}{\sum \mathrm{NS}2_{\mathrm{EI}}+\sum \mathrm{NS}3_{\mathrm{EI}}+\sum \mathrm{Commo}n_{\mathrm{IE}}}$$

The reads of total mRNA was calculated as follows:

(3) $$\mathrm{Total}{\;}_{\mathrm{NS}2}=\sum \mathrm{NS}2_{\;\mathrm{jun}}+\;\sum \mathrm{NS}3_{\;\mathrm{jun}}+\;+\left(P_{\mathrm{NS}2}*\sum \mathrm{NS}2_{\mathrm{EI}}\right)+\left\{(1-p)\sum \mathrm{Commo}n_{\mathrm{IE}}\right\}$$

(4) $$\mathrm{Total}{\;}_{\mathrm{NS}3}=\sum \mathrm{NS}2_{\;\mathrm{jun}}+\;\sum \mathrm{NS}3_{\;\mathrm{jun}}+\;+\left(P_{\mathrm{NS}3}*\sum \mathrm{NS}3_{\mathrm{EI}}\right)+\left\{(1-p)\sum \mathrm{Commo}n_{\mathrm{IE}}\right\}$$

The weighted splicing efficiency of NS2 and NS3 was defined as the propotion: $\mathrm{SE}{\;}_{\mathrm{NS}2\;}=\frac{\sum \mathrm{NS}2_{\;\mathrm{jun}}\;}{\sum \mathrm{Tota}l_{\mathrm{NS}2}}$ and $\mathrm{SE}{\;}_{\mathrm{NS}3\;}=\frac{\sum \mathrm{NS}3_{\;\mathrm{jun}}\;}{\sum \mathrm{Tota}l_{\mathrm{NS}3}}$. All of the SE value from WT and NS100 were normalize to spliced transcripts / unspliced transcrpt.

### Immunoprecipitation (IP)

Cells transiently expressed with the WT NS1 or NS1 variants with fusion tag were lysed with lysis buffer containing proteinase inhibitor cocktail (Roche Diagnostics GmbH, Mannheim, Germany). The whole cell lysates (WCL) were clarified by centrifugation and kept one-tenth of the volume as the input control. Each sample was incubated at 4°C overnight and mixed with 20 µl of Anti-FLAG® M2 Magnetic Beads (Merck KGaA, Darmstadt, Germany) which has been equilibrated by Tris Buffer Saline (TBS). Subsequently, the protein-bead mixtures were washed thoroughly with TBS for five times and the target proteins were eluted in SDS sample dye. The interaction profile was revealed by western blot analysis.

### Measurement of cytokine levels with quantitative real-time RT–PCR (qRT-PCR) or ELISA.

The effect of RG-AIV-WT and NS100 infection on cytokine expression in cells and in the mouse model was evaluated following the method and strategies described in a previous study [24]. First, various cell lines were infected with the indicated virus at an MOI of 1 for 6, 12, and 24 hours. Subsequently, total RNA was extracted from infected cells by Trizol® (Invitrogen, Carlsbad, CA, USA) and processed by the TURBO DNA-Free kit (Invitrogen). Besides, six-week-old BALB/c (5 mice in each group) received 1 × 10^5^ PFU (in 50 μL) of the reassortant viruses by intranasal route. Lungs, were collected from the mice infected at 12 and 24 hpi. The total RNA was extracted from the homogenized lung (0.15 g) by Maxwell RSC simply RNA Tissue kit (Promega). The experimental protocol and sample collection of animals had been approved by the Institutional Animal Care and Committee of National Chung Hsing University (IACUC number: 107-148).

Overall, the expression level of cytokines and viral M segment was estimated by one-step RT–PCR using gene-specific primers following one previous study [24]. In brief, cDNA was synthesized by reverse-transcribing RNA using the GoTaq 1-Step RT-qPCR System kit (Promega), followed by qRT-PCR to detect cytokine levels (CFX Connect™ Real-Time PCR Detection System, Bio-Rad). Data from three independent experiments were analyzed using the 2^-ΔΔ^Cq method with endogenous β-actin as a reference, and all values were expressed as fold changes relative to the mock (non-infection) group. In addition, IFN-β protein concentrations in the serum of each IAV-infected mouse were determined by commercial ELISA (VeriKineTM Mouse Interferon Beta ELISA Kit, PBL Assay Science, Piscataway, NJ, USA) following the manufacturer’s instructions.

### Pathogenicity study in the mouse model

Eight BALB/c mice (five-week-old females) in each group were intranasally inoculated with 10^5^ PFU of the RG-AIV-WT or NS100 viruses. Body weight and survival were monitored daily for two weeks. The experimental protocol was approved by the Institutional Animal Care and Committee of National Chung Hsing University (IACUC number: 107-148).

### Statistical analysis

The growth kinetics between groups was comparatively analyzed by Two-way ANOVA, and the data were displayed as mean ± standard deviation. Differences of cytokine expression levels among groups were evaluated by SAS (SAS Institute, Cary, North Carolina, USA) using the unpaired student t-test. While, the protein ratio of NS2/NS1 or NS2/NP was analyzed by GraphPad Prism 9 software (GraphPad Software, San Diego, California, USA) using an unpaired Student's t-test. The statistical analysis in the pathogenicity study was performed using the method of Kaplan Meier with a Log-rank test. Unless otherwise specified, all data were analyzed using GraphPad Prism 5 software (GraphPad Software, San Diego, California, USA). *P*-values less than 0.05 were considered statistically significant.

## Results

### Identification of the novel truncated NS1 virus, NS100

**Figure 1.**
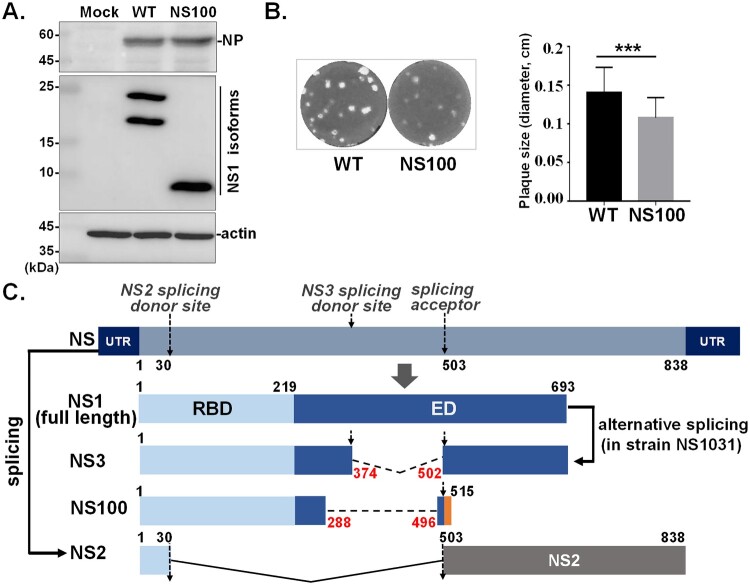
Identification of NS100. (A-B) A virus expressing NS1 variant derived from HPAIV subtype H5N2 (strain NS1031), designated as NS100, was isolated. (A) Wild-type NS1031 (WT) encodes two isoforms (full-length NS1 and NS3 isoforms), while the NS100 protein is smaller than NS3. (B) The plaque size of the NS100 virus is smaller than that of the virus expressing WT NS1. (C) Illustration of the NS segment and the coding regions of the four transcripts derived from it. The NS segment transcribes unspliced NS1 and spliced NS2. NS1 protein has two functional domains: RNA binding domain (RBD) and effector domain (ED). NS1 of H5N2 strain NS1031 also generates NS3 RNA through alternative splicing, with an internal deletion between splicing dinucleotides _374_GU-AG_502_. Sequence analysis indicated NS100 is an NS1 variant containing sequences corresponding to NS segment 1–515 residues with an internal deletion of residues 288-496, while the splicing acceptor dinucleotide (AG_502_) for NS2 is retained in NS100 RNA. The nucleotide numbers indicate the coding region, with the deletion regions in NS3 and NS100 represented by dashed lines and highlighted in red.

A new influenza variant was discovered during the propagation of the RG-AIV-T375G virus in our previous study [24]. Notably, the protein expression pattern of this variant differed from that of the reassortant virus bearing the wild-type NS segment (RG-AIV-WT, or WT in short) (Figure 1(A)). The Average plaques size of the NS100 is slightly smaller than that of the WT virus (Figure 1(B)). It is known that the NS1 gene from the NS1031 strain encodes two NS1 isoforms [24]: the full-length NS1 and the internally deleted NS3 (Figure 1(C)). Surprisingly, both NS1 and NS3 were absent in cells infected with the NS100 virus. Instead, a much smaller protein, approximately 10 kDa, was detected using an NS1-specific antibody.

Sequence alignment confirmed that the NS100 virus has an NS segment with an internal deletion spanning nucleotides 288-496, while the NS2 splicing acceptor dinucleotide (AG at positions 501-502) remains intact (supplementary Figure S1A). Consequently, the NS100 virus expresses a C-truncated NS1 variant that includes amino acids 1–95 of the WT1031 NS1 and five out-of-frame amino acids resulting from genetic misreading (supplementary Figure S1B and Figure 1(C)). Based on the number of amino acids, this novel NS1 variant was designated as NS100.

### NS100 maintains comparable viral growth in different host cells

**Figure 2.**
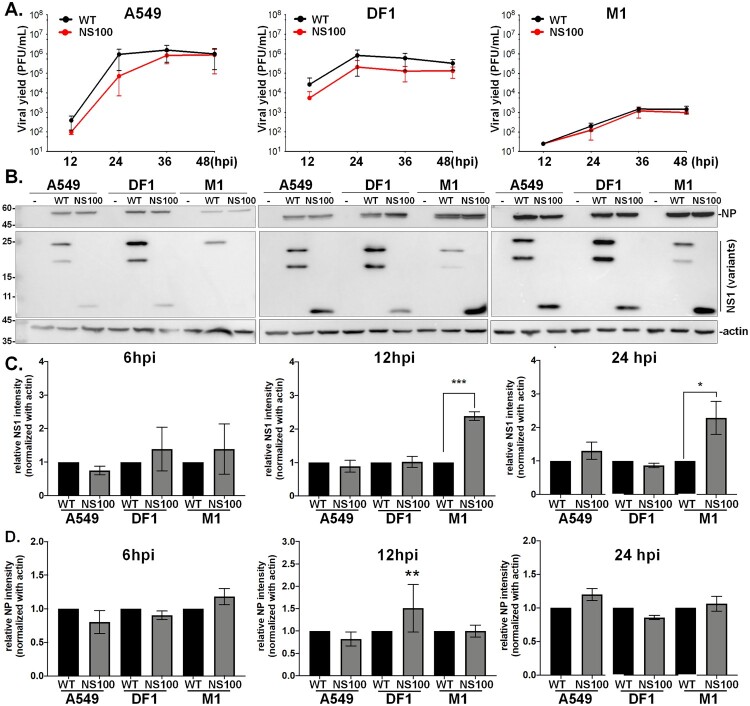
NS100 virus maintains a comparable growth rate as virus expressing WT NS1. (A) Viral growth kinetics of viruses expressing WT NS1031 (RG-AIV-WT, labelled as WT) and NS100 were analyzed in mammalian (A549 and M1) and avian (DF1) cells. Cells were infected at a multiplicity of infection (MOI) of 0.01. Viral yield at 12, 24, 36, and 48 hours post-infection (hpi) was measured by viral plaque assay. (B) Viral proteins at 6, 12, and 24 hpi were detected by western blot analysis. The intensity of viral proteins, including NS1 (C) and NP (D), was quantified using ImageJ. The level of RG-AIV-WT viral protein was arbitrarily set to 1, and the relative expression patterns of NS1 and NP were calculated and plotted. Values represent mean ± SEM. ***P < 0.001 (unpaired *t*-test).

Next, we characterized the viral growth kinetics of RG-AIV-WT (WT) and NS100 in two mammalian cell lines (human A549 and mouse M1) and avian DF-1 cells. Surprisingly, NS100 exhibited a growth rate similar to RG-AIV-WT across all cell lines, indicating that the truncated NS1 protein did not impact viral replication *in vitro* (Figure 2(A)). Interestingly, despite H5N2 (strain 1031) being initially isolated from chickens, the replication of both viruses showed comparable growth advantages in A549 and DF-1 cells. Notably, the viral load of both viruses in M-1 cells was relatively lower than in the other two cell types.

We further investigated the expression of the viral proteins NP and NS1 in infected cells (Figure 2(B,C)). Consistent with the growth kinetics results, the expression pattern of NP proteins was similar between NS100 and RG-AIV-WT in human, chicken, and mouse cell lines at 6, 12, and 24 hours post-infection (hpi) (Figure 2(B)). Additionally, the accumulated NS1 proteins were comparable between NS100 and RG-AIV-WT in human and avian cells, while NS100 displayed more robust NS1 expression in mouse cells compared to RG-AIV-WT.

The deletion of the ED region in NS1 did not affect NS100 viral replication across various host cell types, suggesting that NS100 may utilize a unique infection mechanism that compensates for the extensive truncation in its NS1 region.

### NS100 inhibits cytokine production in infected cells

**Figure 3.**
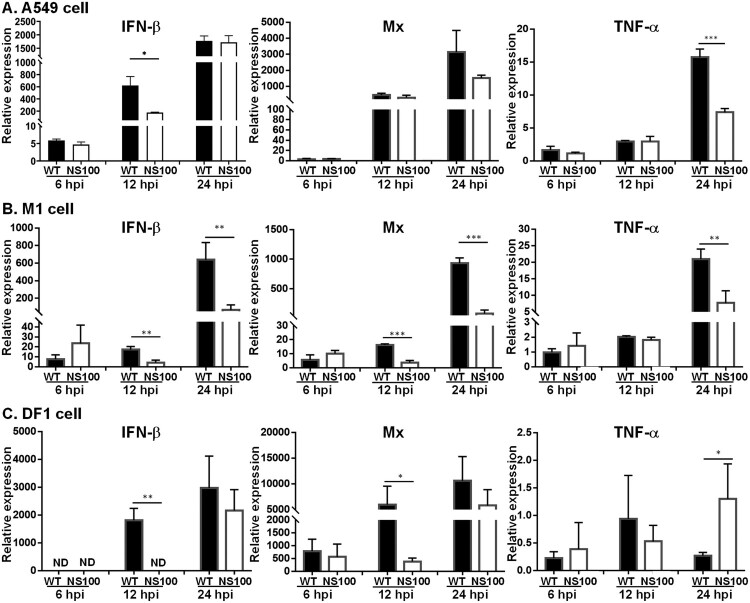
Expression profiles of cytokines in mammalian or avian cells infected with RG-AIV-WT, or NS100 virus. Cytokine expression profiles were analyzed in mammalian A549 (A), murine M1 (B), and chicken DF1 (C) cells infected with AIVs (WT or NS100) at an MOI of 1. Total RNA was harvested at 6, 12, and 24 hpi. The expression levels of cytokines, including IFN-β, Mx, and TNF-α, were detected by real-time RT-PCR and normalized to levels in uninfected cells. All experiments were performed in triplicate. Significant differences between the two groups are denoted by *, **, *** indicating *p* < 0.05, < 0.01, < 0.001, respectively, comparing the particular virus with others.

NS1 modulates cytokine expression that facilitates the establishment of the early stage of viral infection[12,13]. Subsequently, the expression of cytokines, including IFN-β, Mx, and TNF-α in cells infected with three types of cells was evaluated by real-time RT–PCR. Compared with the mock treatment, viral infection indeed elicited expressions of these cytokines (Figure 3). Moreover, in general, cytokine levels in cells infected with the NS100 virus was lower than those infected with RG-AIV-WT; for instance, RNA levels of IFN-β, Mx, and TNF-α are significantly lower in M-1 cells infected with NS100 virus than in the RG-AIV-WT virus (Figure 3(B)). Similarly, a lower TNF-α RNA was accumulated in A549 cells infected with NS1 virus compared with that infected with RG-AIV-WT virus (Figure 3(A)). Of note, in DF-1 cells, the virus expressing NS100 stimulated a higher level of TNF-α RNA than WT NS1 at 24 hpi, although IFN-β and Mx levels remained lower levels under NS100 virus than RG-AIV-WT virus infection at 12 hpi (Figure 3(C)).

### NS100 maintains pathogenicity in mice

**Figure 4.**
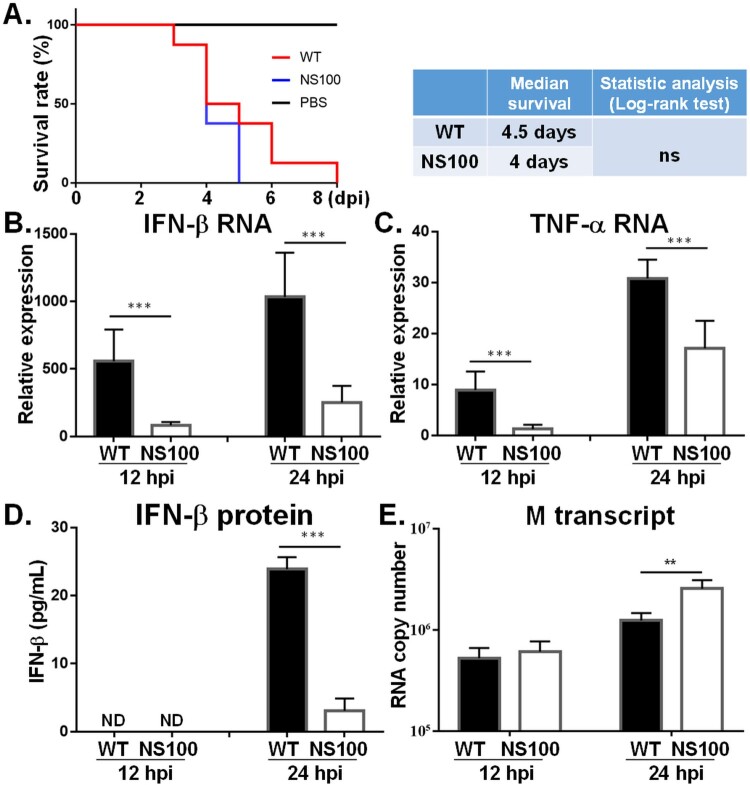
Survival rate and expression profiles of cytokines and viral RNA in mice infected with various AIVs. (A) The survival rate of mice under infection. Mice (n = 8) were infected with AIVs. The survival rates of infected (WT or NS100 virus) and control mice (PBS) were monitored for 14 days post-infection (dpi). (B-C) The cytokine RNA level in the blood of infected mice. To evaluate cytokine expression levels, blood was collected from mice (n = 5) at 12 and 24 hpi. The RNA levels of cytokines, including IFN-β (B) and TNF-α (C), were detected by real-time RT-PCR. Moreover, the IFN-β protein level in blood was detected by ELISA (D). Additionally, mice were sacrificed at 12 and 24 hpi and lung samples were collected for detection of viral RNA (M gene) level (E). All experiments were conducted in triplicate. Significant differences between the two groups are denoted by *, **, ***, indicating *p* < 0.05, < 0.01, and < 0.001, respectively, comparing the particular virus with others.

NS1 is regarded as one of the determinants of influenza virulence, and hence attempt was made to depict the pathogenicity of NS100 virus by monitoring the overall survival rate. As shown in Figure 4(A), in the RG-AIV-WT infection group, the last mouse died on the eighth day post-infection (dpi), whereas no mice infected with NS100 survived beyond 5 dpi. Despite this, survival curve analysis showed no significant difference between the two infection groups. (Figure 4(A), left panel). Moreover, the mean survival time of mice infected by the RG-AIV-WT virus is 4.5 days, while the NS100 virus is 4 days (Figure 4(A), right panel). Together, these results indicate that the influenza virus expressing C-terminal truncated NS100 protein retains similar pathogenicity in the mouse model.

We further monitored cytokine levels and viral RNA loads in the infected mice. Consistent with the finding in mouse M1 cells, RG-AIV-WT robustly elicited cytokines in the mouse model (Figure 4(B,C)). At both 12 hpi and 24 hpi, mice infected with RG-AIV-WT, both IFN-β (Figure 4(B)) and TNF-α mRNA (Figure 4(C)) in the lung, as well as the protein level of IFN-β (Figure 4(D)) in blood, were significantly higher than those infected with the NS100 viruses (Figure 4(B,C)). Moreover, a significantly higher level of the viral M transcript was detected in the lungs of mice infected with the NS100 virus than in those infected with RG-AIV-WT, indicating an efficient infection.

### NS100 interacts with PKR: The N-terminal 95 residues play a crucial role in inhibiting PKR function

**Figure 5.**
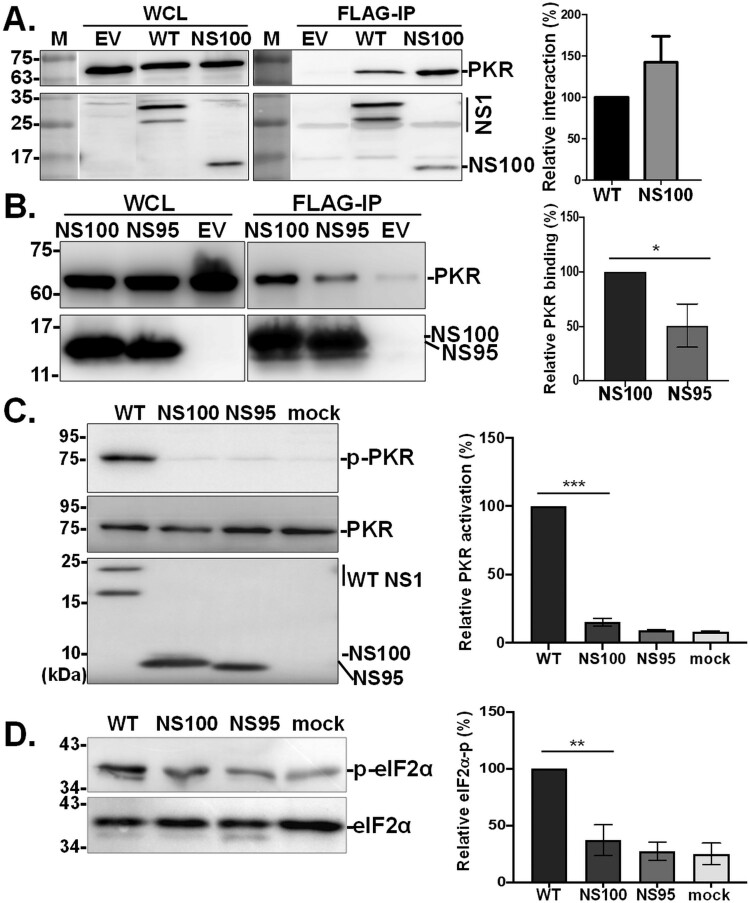
Effects of NS100 on interaction and phosphorylation of protein kinase R (PKR). (A) Interaction of NS1 derived from NS100 or WT virus with cellular PKR was analyzed via co-immunoprecipitation (Co-IP) in HEK293 T cells transfected with plasmids encoding NS gene fused with FLAG-tag. (B) Both NS100 and NS95, the protein harbouring the N-terminal 95 amino acid of NS100, are able to interact with PKR. (C) The effect of NS1 variants (NS100 or NS95) on PKR activation was determined by infecting A549 cells with RG-AIV-WT (WT), NS100, or RG-AIV-NS95 viruses at 0.5 MOI for 24 hours. PKR and phosphorylated PKR (p-PKR) were detected with p-PKR/PKR ratio estimated. (D) Downstream signalling of PKR was monitored by detecting eIF-2α. Relative PKR (or eIF-2α) phosphorylation levels were compared with mock samples, arbitrarily set as 1. Values represent mean ± SEM from three repeats. *, **, *** indicate significance at *p* < 0.05, *p* < 0.01, and *p* < 0.001, respectively, using one-way ANOVA statistical analysis.

Evidence from both *in vitro* and *in vivo* models shows that NS100 infection efficiency is comparable to the virus expressing WT NS1, while overall cytokine levels are lower (Figures 3 and 4). NS1 is crucial in dampening IFN-mediated antiviral defenses, particularly by physically interacting with and suppressing phosphorylation of PKR as well as its downstream antiviral activations. It is possible that NS100 may retain the characteristics of NS1 in this aspect. To investigate this, we first assessed the influence of the C-truncated version of the NS1 on PKR interaction by transiently expressing various NS1 variants (WT or NS100) by co-immunoprecipitation (Co-IP) approach. Surprisingly, despite the deletion of the extended ED region, NS100 pulled down PKR is at least equivalent to, if not more effective than, WT (Figure 5(A)).

Given that NS100 comprises the 1–95 residues of NS1 followed by five additional residues (DSRTC) added to the C-terminus through translational misreading, it is intriguing to explore whether these 95 residues in NS100 are primarily responsible for its regulatory function observed in our analysis systems. Using the Co-IP approach, it is noteworthy that NS95-FLAG, the protein without DSRTC, is able to interact with PKR, although to a lesser extent as compared to NS100-FLAG (Figure 5(B)).

Structurally, the interaction of NS1 with PKR prevents PKR activation, thereby reducing the effect of PKR on the phosphorylation of its substrate, eIF2α protein, which leads to the shutdown of global translation [40,41]. The significant interaction of the C-truncated NS1 proteins with PKR prompted us to further investigate PKR activation levels in the course of NS100 infection. While RG-AIV-WT virus infection induced phosphorylation of PKR in A549 cells, infections with NS95 and NS100 viruses significantly reduced PKR phosphorylation to levels comparable to those observed with mock treatment (Figure 5(C)). Notably, the activation status of PKR is reflected in the phosphorylation level of eIF2α, the substrate of PKR (Figure 5(D)). These results suggest that NS100 virus might exploit its more potent inhibitory effect on the PKR pathway to facilitate infection.

### NS100 is able to bind dsRNA

**Figure 6.**
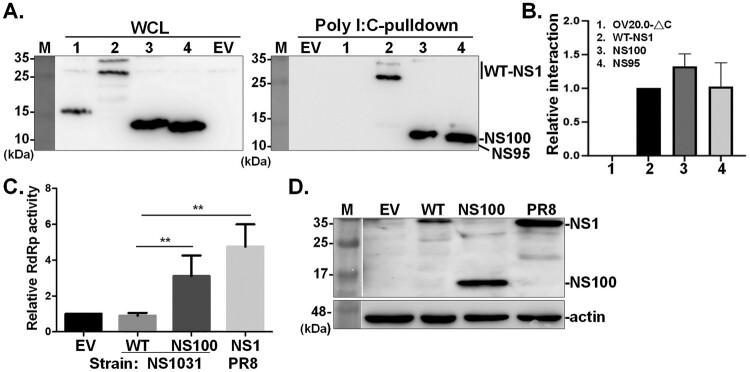
Evaluation of the RNA binding ability and effect on viral genome replication of NS1 and NS100 protein. RNA binding ability of NS100 and NS95 was determined by a poly(I·C) pulldown assay(A-B). Total cell lysates from cells transfected with FLAG-empty vector (lane EV), OV20.0-ΔC (lane 1), WT-NS1 (lane 2), one of the NS1 variants including NS100 (lane 3) or NS95 (lane 4) were incubated with agarose beads coated with synthetic dsRNA poly(I:C). EV and OV20.0-ΔC serve as the negative control for dsRNA binding. The amount of NS1 proteins bound to poly(I:C) agarose beads was eluted and detected via western blot analysis. Relative dsRNA binding levels between groups were plotted and normalized to WT-NS1, which was arbitrarily set as 1(B). The effect of NS100 on viral RNA-dependent RNA polymerase (RdRp) activity was assessed using a mini-genome assay (C-D). The NS1 protein from H1N1 (strain PR8) served as a positive regulator of viral RdRp function. Luciferase expression, driven by the influenza viral promoter, was used as a readout of RdRp activity (C). The expression levels of NS1 and NS100 were confirmed by Western blot analysis (D). Relative RdRp activity was compared with the sample without NS1 (empty vector, EV), arbitrarily set as 1. Values represent mean ± SD from three repeats. **indicate significance at *p* < 0.01, using one-way ANOVA statistical analysis.

Several functional-structural studies revealed that NS1 forms a homodimer by dimerizing RBD and ED with the matching domains of another NS1 monomer, and this dimerization affinity is mainly facilitated by interactions between RBDs [15,42,43]. On the other hand, via RBD, NS1 binds to dsRNA, which in turn prevents the IFN induction and PKR activation. The comparable ability of NS1 variants on PKR binding prompted us to evaluate the known intrinsic functions of NS1. We first investigated whether NS100 and NS95 exhibit dsRNA binding ability, which is required for the formation of dimeric NS1. In this approach, in addition to the empty vector (EV), ORFV OV20.0, another RNA binding protein [29,30], with deletion of the entire dsRNA binding domain (OV20.0-ΔC, in lane 1), serves as the negative control. As evidenced by the pulldown assay using the synthetic dsRNA, poly(I·C), NS1 and the two NS1 variants (NS100 and NS95) harboured dsRNA binding ability (Figure 6(A)). Notably, the dsRNA binding ability of NS100 and NS95 is comparable to that of NS1031 (Figure 6(B)). The dsRNA-binding activity of NS100 was further confirmed by a competitive poly I:C pull-down assay. Pre-incubation of NS100-containing lysate with 0.4 μg free poly I:C substantially reduced NS100 capture by poly I:C-conjugated beads, demonstrating specific binding to dsRNA (supplementary Figure S2), indicating that the RBD alone is sufficient for dsRNA interaction.

### NS100 enhances viral RNA genome replication

The NS1 protein is known to associate with viral ribonucleoprotein complexes (vRNPs) via its RNA-binding domain [44]. Previous studies have shown that NS3, an internally truncated variant of NS1031, rather than the full-length NS1031, acts synergistically with the viral RNA-dependent RNA polymerase (RdRp) complex [24]. To further investigate whether NS100 can regulate viral RdRp function, we performed a mini-genome assay. As expected, NS1 derived from the PR8 strain significantly enhanced genome replication activity, as indicated by increased reporter gene expression (Figure 6(C)). In contrast, co-expression of NS1031 protein (Figure 6(D)) failed to stimulate RdRp activity, showing luciferase levels comparable to the mock control. Notably, NS100 was also able to enhance RdRp activity (Figure 6(C)), suggesting that the truncated variant retains this key functional property.

### NS100 protein retains dimer formation ability

**Figure 7.**
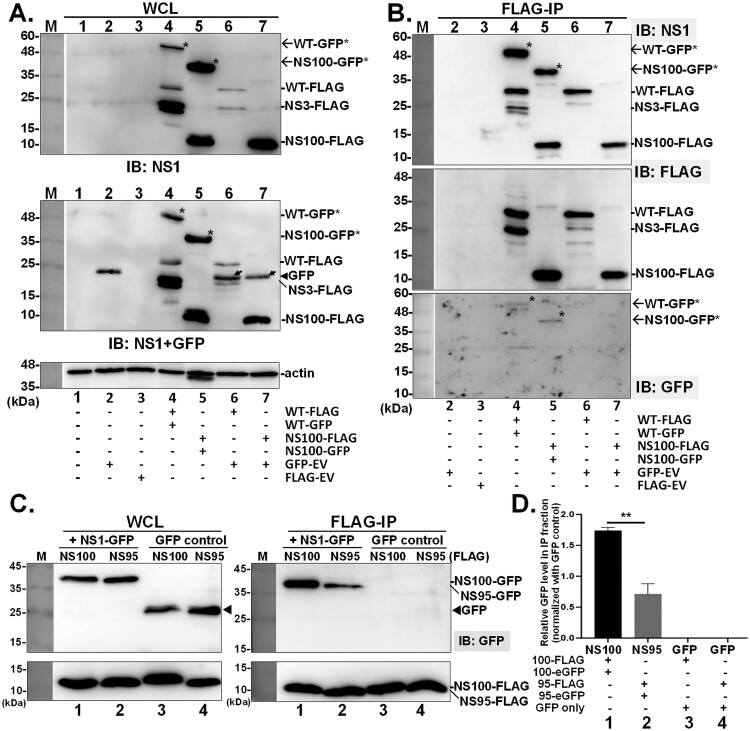
Analysis of the dimer formation ability of NS100. (A-B) Dimer formation of NS100 was analyzed by co-expressing NS variants fused with different tags (either FLAG or GFP) in HEK293 T cells. Co-transfection of FLAG-tagged NS with the GFP empty vector (GFP-EV) was included as a negative binding control. After 24 hours of transfection, whole cell lysate (WCL) was harvested for immunoprecipitation with FLAG-antibody beads (A). The arrowhead indicates the GFP protein. (B)The GFP signal in fractions pulled down by FLAG-beads, serving as an indicator of dimeric NS1 formation, was detected by immunoblot analysis using various antibodies (i.e. IB: NS1, FLAG, eGFP). The asterisks indicate GFP-tagged NS1 and NS100. Subsequently, the comparative dimerization ability of NS100 and NS95 proteins was determined by the same system (C). The GFP-tagged NS protein in the IP fraction, indicating the dimer level, was estimated and normalized to its GFP vector control group, which was arbitrarily set as 1(D). Experiments were conducted in three independent repeats, and ** indicates not significant (*p* > 0.01).

Next, dimerization of WT NS1 and NS100 was determined by co-expression of NS1 fused with two different tags, i.e. FLAG and GFP proteins in cells, followed by Co-IP using beads conjugated with the FLAG-specific antibody. Thus, the GFP fusion protein signals (indicated as asterisks) appearing in the IP fraction will provide evidence for the presence of dimerized NS1 (Figure 7(B)). In this set of experiments, GFP moiety expressed from empty vector (GFP-EV) serves as the negative control for NS1 interaction (e.g. the lane 6 and lane 7 in Figure 7). As shown in Figure 7(B), WT-FLAG interacted with WT NS1 fused with GFP tag (lane 4 with asterisk in Figure 7(B), top panel with NS1 antibody). Similarly, NS100-FLAG successfully pulled down NS100-GFP, indicating the dimer formation (lane 5 with an asterisk in Figure 7(B), top panel). While, GFP was not associated with either WT-FLAG or NS100-FLAG (lane 6 and lane 7 in Figure 7(B), the bottom panel). Subsequently, we investigated whether the N-terminal 95 residues in NS100 are sufficient for homodimerization. Co-immunoprecipitation experiments showed that GFP-tagged NS95 was pulled down by NS95-FLAG, indicating dimer formation, albeit to a lesser extent than that observed with full-length NS100 (Figure 7(C,D)). Furthermore, dimer formation of NS1 proteins were further confirmed by formaldehyde cross-linking approach [45]. As with NS1 derived from PR8 strain, NS1031 and its deletion variants, including NS100, NS95, and the RBD fragment are able to form dimer (as indicated as asterisk signs in supplementary Figure S3). In summary, NS100 forms dimers and retains the dsRNA binding abilities *in vitro*.

### The splicing of NS transcript is inefficient in the NS100 virus

**Figure 8.**
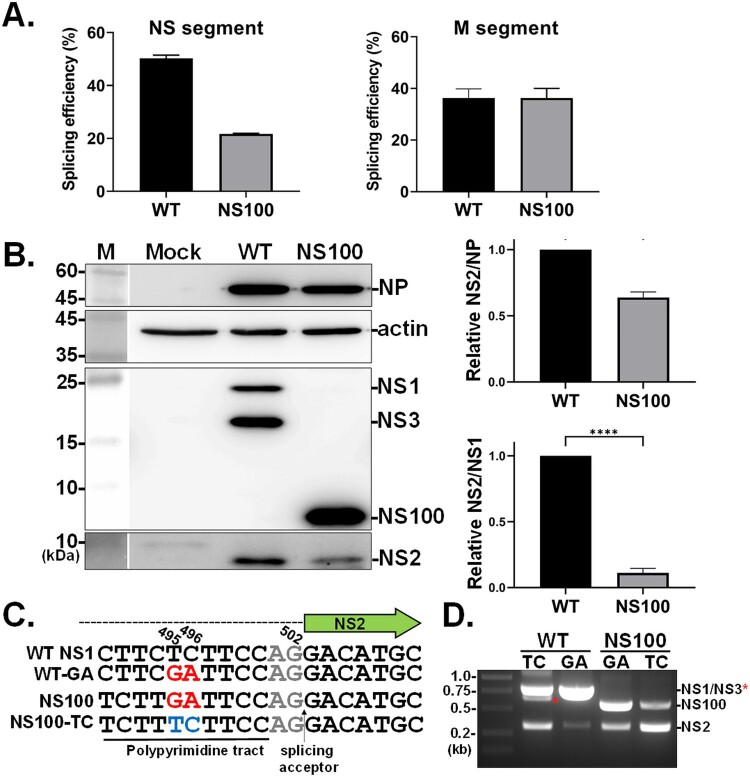
The level of NS2 RNA and protein expressed from NS100 virus. A549 cells were infected with RG-AIV-WT (WT) and NS100 viruses at 0.5 MOI for 12 hpi. Splicing efficiency of the two viral segments, including M and NS genes, in cells infected with WT and NS100 virus was estimated based on the NGS approach (A). Moreover, the viral protein profile, including NS1, NS1 variants (NS100, NS3), NS2, and NP in infected cells, was determined by western blot analysis (B). The relative NS2 level was normalized with NS1 or NP protein and was estimated and plotted, with the ratio in RG-AIV-WT arbitrarily set as 1. Values represent mean ± SD from three repeats. ***, **** indicate significance at *p* < 0.001, *p* < 0.0001 using unpaired t-test statistical analysis. (C) The influence of residues GA (495-496) within the predicted polypyrimidine tract on NS100 transcript was further investigated using a site-directed mutagenesis strategy, where GA nucleotides were reconstituted to TC (NS100-TCF), while TC on WT NS1 was changed to GA (WT-GA). (D) The splicing level of the four genotypes was evaluated by the mini-genome approach, including the plasmid-bearing mutated sequences on the NS segment.

It is well established that the NS segment transcribes two major RNAs, the unspliced NS1 and NS2, via an alternative splicing mechanism. However, in the H5N2 strain NS1031, the NS1 RNA undergoes additional splicing, resulting in the production of the NS3 variant [24]. Interestingly, validation of full-genome sequences by next-generation sequencing (Illumina platform) revealed an unexpected finding: as estimated using a previously described method [39], the spliced/unspliced ratio of the NS segment was significantly lower in the NS100 virus compared to the wild-type (WT) virus (Figure 8(A), left panel). Notably, this phenomenon was not observed in the M transcript, another gene segment known to undergo splicing (Figure 8(A), right panel). Furthermore, the level of the NS2 protein was reduced in the NS100 virus, correlating with the lowered NS2 transcript levels (Figure 8(D)). When NS2 expression was normalized to viral NP or NS1 levels, the NS100 virus exhibited a proportionally lower amount of NS2 compared to the RG-AIV-WT virus (Figure 8(B), right panels).

Subsequently, factors influencing NS2 splicing efficiency were further investigated. Notably, sequence alignment of the NS fragment from the WT and NS100 viruses revealed polymorphisms upstream of the 3’ splice site AG dinucleotide (at residues 501-502) (Figure 8(C), left). Specifically, a transversion of the GA dinucleotide was observed at residues 495–496 in NS100, whereas the TC dinucleotide, a pyrimidine nucleotide, was conserved in WT NS1. Given the position and sequence characteristics, this variation is likely located within the predicted polypyrimidine tract (PPT), a cis-element critical for splicing. To assess whether the GA transversion contributes to the reduced NS2 splicing in NS100 RNA, we generated a construct (i.e. NS100-TC) that replaced the GA with the pyrimidine TC in the NS100 gene. Using the mini-genome assay, NS segment transcription was monitored. As shown in Figure 8(D), the yield of the NS2 transcript was significantly increased when GA was substituted with TC in the NS100 transcript, indicating the TC dinucleotide restores splicing efficiency. In contrast, when the WT NS segment (WT-GA), bearing mutations that mimic the GA variation seen in NS100, was tested, splicing of both NS3 and NS2 was strongly diminished. These results suggest that the GA variation may disrupt the function of the PPT.

### NS100 infection induced a lower level of anti-viral response in A549 cells

Moreover, to investigate the possible mechanism of growth advantage of viruses carrying C-truncated NS1 protein, the comparative transcriptome analysis was conducted to monitor the global gene expression profiles in infected A549 cells.

**Figure 9.**
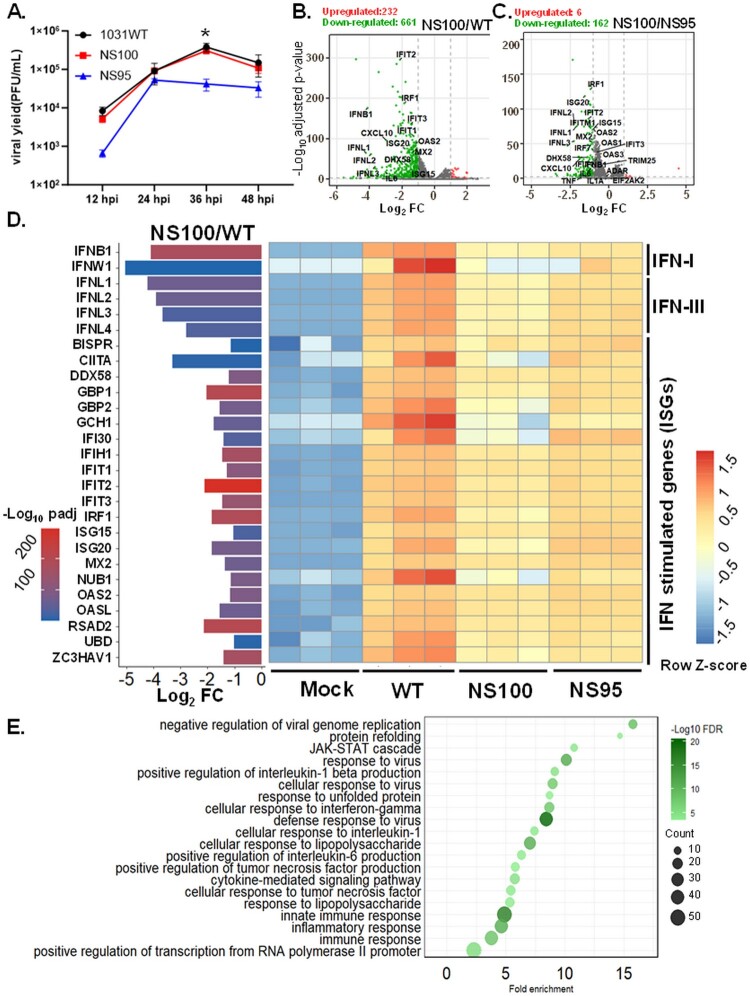
Comparative growth kinetics and transcriptomic analysis of viruses expressing various NS1 proteins. A549 cells were infected with either RG-AIV-WT, NS100, NS95 virus at MOI of 0.5, or left untreated. Viral yield at 12, 24, 36, and 48 hours post-infection (hpi) was measured by viral plaque assay (A). (B-C) Differentially expressed genes (DEGs) were identified using a volcano plot. Two comparison groups were performed: (B) NS100 vs. WT, (C) NS100 vs. NS95. The red and green dots represent upregulated genes and down-regulated genes determined by adjusted *p*-value (padj)< 0.01 with Log2 FC ≥ 1 or ≤ −1, respectively. The gene labels indicate pro-inflammatory cytokines and interferon (IFN)-stimulated genes (ISG). (D) Expression profiles of IFN, including type I and Type III (IFN-I, IFN-III, respectively), and ISGs were visualized by heatmap with hierarchical clustering. The infected group (WT and NS100) and the untreated group (Mock) are both represented in triplicate. Log_2_ read counts are transformed into Z-scores for each gene. (E) Functional enrichment analysis represented the top 20 Gene Ontology biological processes of down-regulated genes in the comparison group of NS100 vs. WT.

Initially, the comparative growth kinetics of the three viruses were examined. Overall, NS95 exhibited moderately reduced viral yields compared with NS100 and the wild-type virus. Notably, at 36 hpi, the NS95 virus produced significantly fewer viral progeny (Figure 9(A)). Subsequently, RNA-seq analysis was performed. Volcano plots illustrated distinct profiles of differentially expressed genes (DEGs) between cells infected with the NS100 virus and those infected with RG-AIV-WT or NS95. In general, the levels of induced DEGs in NS100-infected cells were lower than those in the RG-AIV-WT group (Figure 9(B)) and the NS95 group (Figure 9(C)). Consistently, gene expression profiles visualized by heatmap revealed that RG-AIV-WT virus upregulated the expression of several genes involved in the innate immune response, including those associated with IFN signalling, such as type I (IFN-B1, IFN-W1) and type III IFNs (IFNL1-L4), as well as 20 ISGs, including DDX58 (RIG-I), MX2, OAS1, OAS2, OASL, ISG15, ISG20, IFIT1, IFIT2, IRF1, etc., at higher levels than the NS100 virus (Figure 9(D)). To confirm these transcriptomic observations, IFN-β and ISG15 were quantified by real-time RT–PCR, with data provided in supplementary Figure S4.

Moreover, functional enrichment analysis was performed to explore the regulatory impact of NS100 infection on the cellular response. The top 20 Gene Ontology biological processes of downregulated genes in the comparison between NS100 and WT virus are summarized (Figure 9(E)). Interestingly, most of the downregulated DEGs in the NS100 group were involved in the negative regulation of viral genome replication, protein refolding, and various biological processes related to antiviral responses, suggesting that NS100 infection globally reduces the host antiviral response.

## Discussion

In the present study, we identified and characterized a naturally occurring NS1 variant with a C-terminal deletion, referred to as the NS100. The NS100 virus encodes an NS1 protein composed of N-terminal 1–95 residues identical to those of wild-type (WT) NS1 (derived from strain NS1031 of subtype H5N2), followed by five additional amino acids (DSRTC) through translational misreading. To our knowledge, this is the first report to demonstrate that an influenza A virus with a significantly truncated NS1 retains comparable virulence to the WT virus.

The influenza viral genome is compact, containing essential genes for replication [46], and deletions or truncations typically lead to virulence attenuation. While the N-terminal RBD domain is crucial for NS1 function, several studies show that C-terminal truncations of NS1 in influenza A virus (IAV) often reduce pathogenesis in various hosts [22,47-52]. For example, reassortant PR8 variants expressing truncated NS1 (124, 80, and 38 amino acids) had lower viral yields compared to the wild-type [53]. A similar trend was observed with the swine IAV (TX/98 strain) where C-terminally truncated NS1 variants expressing residues 1-73, 1-99, and 1–126 exhibited reduced viral titres [50]. However, unlike H1N1, attenuation in the TX/98 virus did not correlate with NS1 length, as the shortest NS1 (1-73) was more virulent than the 1–126 variant. On the other hand, to establish a new replication niche, NS1 is susceptible to adaptive mutations, including point mutations or deletion, which enhance the fitness of the influenza virus within hosts, as evidenced in two swine influenza viruses [54]. Additionally, an internal deletion of the NS1 isoform (designated NS3, missing residues 126-168) enhances viral growth in various host cells. Specifically, NS3 from the human H3N2 virus aids viral adaptation in mice [25], while NS3 from avian H5N2 enhances replication in mammals [24]. Overall, while the link between NS1 and virulence is well-established, the correlation between NS1 length and pathogenicity can vary by virus strain and host range. In the current study, NS100 virus, a reassortant PR8 virus containing NS segment originating from avian influenza (strain NS1031, subtype H5N2), was isolated from MDCK culture. Notably, in our study, the novel C-truncated NS100 variant unexpectedly replicated efficiently in cells (Figure 2(A), Figure 4(E)) demonstrated a survival rate in mice comparable to that of the wild-type (WT) virus (Figure 4(A)). Although NS100 demonstrated enhanced viral RdRp activity *in vitro* (Figure 6(C)), this did not translate into significantly increased viral growth or virulence in cell culture or animal models. This discrepancy likely reflects the complexity of the in vivo environment – such as immune pressures, tissue architecture, and multicellular interactions – that constrain proportional increases in viral replication. Collectively, these findings suggest that the partial deletion of the ED domain in NS1 does not impair viral fitness, possibly due to viral adaptation in the heterogeneous host or the sufficiency of RBD-mediated functions in supporting viral fitness.

It is well documented that NS1-driven viral fitness relies on antagonizing the IFN-mediated host antiviral response [12,13]. As indicated in Figures 4 and 9, as with the virus expressing WT NS1, NS100 infection triggered an antiviral response. Interestingly, comparative transcriptome analysis revealed that compared to the WT virus, cells infected with NS100 exhibited lower RNA levels of IFN, IFN-stimulated genes (ISGs), and proinflammatory cytokines (Figure 9(C,D), supplementary Figure S4). Additionally, as summarized in Figure 9(E), genes downregulated in NS100-infected cells were primarily associated with antiviral defense mechanisms, including the innate immune response, IFN production, cytokine expression (IL-1β, IL-6, TNF), and the JAK/STAT signalling pathway. Notably, these pathways are the main targets of NS1, through which it antagonizes the antiviral response [16]. These findings suggest that NS100 suppresses the antiviral response to a greater extent than the WT virus, which could represent one of the mechanisms underlying the growth advantage of the NS100 virus.

NS1 exerts its modulatory function mainly via interaction with a multitude of host cell factors [12,13]. Since ED provides the interface for the association of NS1 with numerous cellular proteins, it could be argued that the deletion of residues 96–230 may influence the regulatory role of NS100 on the antiviral response. It is known that dsRNA binding and homodimer formation are essential for exerting the IFN antagonistic function [55]. As shown in Figure S1, NS100 retains the full RBD, with conserved R38 and K41 for dsRNA binding. Poly I:C pull-down assay confirmed that NS100 is able to bind the dsRNA (Figure 6). Notably, the dsRNA binding ability of NS95 is comparable to that of NS100, indicating that the RBD alone is sufficient for dsRNA interaction, and the presence of the DSRTC residues in NS100 does not significantly enhance this binding (Figure 6(B)). Although the region required for the formation of NS1-homodimer is not well documented, by the strategy of co-expression of NS100 proteins fused with two different tags (Figure 7), NS100e-GFP protein was co-immunoprecipitated with NS100-FLAG that reveals the formation of NS100-dimer and that was further confirmed by cross-linking approach (supplementary Figure S3). Moreover, NS1 limits the activation of PKR, the major IFN-stimulated gene [56,57], leading to global translational shut-down via phosphorylating elF2α, thereby restricting viral protein production in infected cells [23,58]. Residues 123–127 of NS1 have been reported to play a critical role in PKR interaction and auto-phosphorylation [40,41]. However, our data demonstrated that, despite the absence of residues 123-127, NS100 retains the ability to interact with PKR, as evidenced by similar levels of PKR pull-down compared to WT (Figure 5(A)), and continues to inhibit PKR and eIF2α phosphorylation. It is worth noting that one recent study reported that, in addition to residues 123–127 of the ED, the N-terminal residues R35 and R46 of RBD also play a role in the direct binding of NS1 to PKR, blocking its activation [59]. Given that PKR activation requires a dsRNA inducer, the conservation of the key residues (R35, R38, K41, R46) for binding both PKR and dsRNA (supplementary Figure S1) could enable NS100 to interact with PKR and sequester dsRNA, likely explaining its continued ability to counteract PKR activation. Consistent with this notion, a previous study demonstrated that a truncated NS1 fragment comprising only the N-terminal 82 amino acids (NS82), corresponding to the RBD, exhibited stronger RNA-binding activity than full-length NS1 and inhibited PKR activation [56]. In this regard, the presence of the RBD appears critical, as removal of the C-terminal ED may expose or structurally favour key residues within the RBD, thereby enhancing PKR binding and boosting the antagonistic activity of NS1 against host antiviral defenses. However, structure-based functional analyses are required to strengthen this hypothesis.

In our study, compared to the WT virus, NS100-infected cells displayed lower ratios of both NS2/NS1 mRNA (Figure 8(A)) and protein (Figure 8(B)), indicating a decreased NS2 mRNA splicing efficiency. Site-directed mutagenesis confirmed that sequence variations in the potential polypyrimidine tract of NS100 were responsible for the inefficient splicing of NS2 transcripts (Figure 8(C,D)). It has been documented that the splicing efficiency of the NS segment in IAV may be a key determinant of viral replication and adaptation to new hosts. For instance, the NS segment of A/Brevig Mission/1919/1 (H1N1) is inefficiently spliced compared to other influenza viruses, resulting in elevated levels of NS1 [60]. This splicing inefficiency has been proposed to contribute to the high pathogenicity of the 1918 Spanish influenza pandemic. Additionally, a reduced NS2/NS1 mRNA ratio has been suggested as a viral adaptation mechanism for avian influenza viruses to replicate efficiently in mammals [61]. The avian H7N9 virus contains a unique G540A substitution within the predicted exonic splicing enhancer in the second exon of the NS segment, which affected NS2 mRNA splicing efficiency and enhanced H7N9 viral replication in mammalian cells. These findings suggest that modulation of NS mRNA splicing, leading to increased levels of functional NS1, plays a critical role in viral replication efficiency and host adaptation. However, the precise underlying mechanisms require further investigation.

We acknowledge the limitations of the current study. Our primary objective was to dissect how NS100, which lacks most of the ED, retains infectivity or acquires immune-evasion capacity, with emphasis on RBD-mediated mechanisms, including PKR interaction. Structural modelling suggests that the DSRTC segment in NS100 does not adopt a discrete fold but may act as a flexible tail capable of transient interactions; targeted point-mutation and functional analyses could further clarify its precise contribution. While the ED typically mediates a broad range of host-factor interactions that contribute to antiviral suppression, our findings indicate that NS100 may instead leverage alternative pathways or underappreciated functional plasticity of the RBD. Given the complexity of host antiviral networks and the multifunctional nature of NS1, future studies incorporating interactome mapping and expanded mechanistic analysis will be essential to fully define the strategies by which NS100 antagonizes host immunity.

In conclusion, our study identifies a novel, naturally occurring influenza virus variant carrying a genetic deletion that results in a truncated NS1 protein. The efficient replication of the NS100-containing virus, despite the absence of the ED region, underscores the sufficiency of RBD-mediated functions in maintaining viral fitness. This finding highlights the modular nature of NS1 and raises the possibility that certain naturally occurring truncations may confer selective advantages under specific host or cellular conditions.

We further demonstrate that the replication advantage of NS100 likely stems from altered NS segment splicing efficiency and the retention of a functional RBD, which is essential for enhancing viral RdRp activity, homodimer formation, and dsRNA binding – key features for modulating the PKR pathway. Nonetheless, given the multifunctionality of NS1, the precise molecular mechanisms through which it suppresses host antiviral responses warrant further investigation. Future studies employing omics-based and proteomic approaches will be instrumental in elucidating the dynamic and multifaceted interactions of NS1 variants within the host environment.

## Author contributions

RST, JYT, YTC and CYT: Data curation, Validation, Formal analysis. GRL, RA, CYK and RLK: Validation. WLH: Writing, Conceptualization, review & editing, Supervision, Methodology, Funding acquisition. SCO: Supervision, Methodology, editing.

## Supplementary Material

Fig_S3.tif

Fig S1.tif

Fig_S4.tif

Fig_S2.tif
