# Supplementary figures and images for: A naturally occurring NS1 variant with effector domain deletion gains growth advantages in influenza virus infection
Source: Emerg Microbes Infect. 2025 Sep 3;14(1):2556731. doi: 10.1080/22221751.2025.2556731 (PMC12456053; doi:10.1080/22221751.2025.2556731)

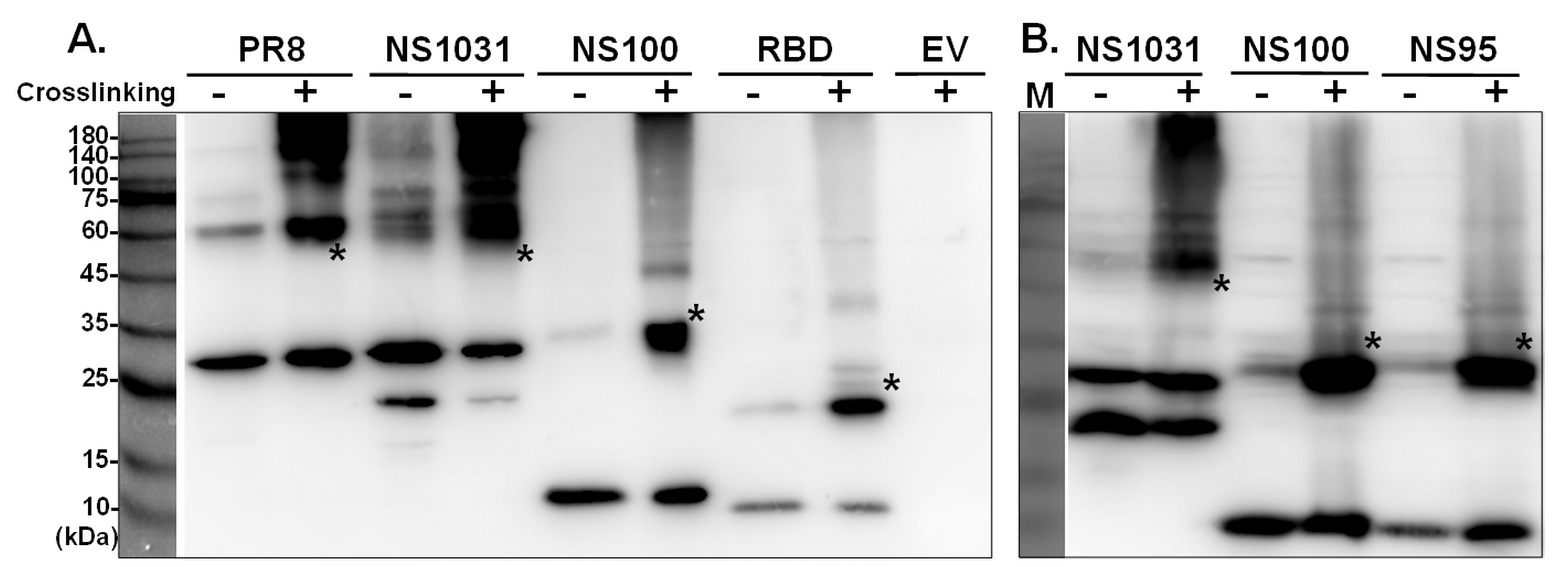

Supplement: Fig_S3.tif [file TEMI_A_2556731_SM4357.tif]

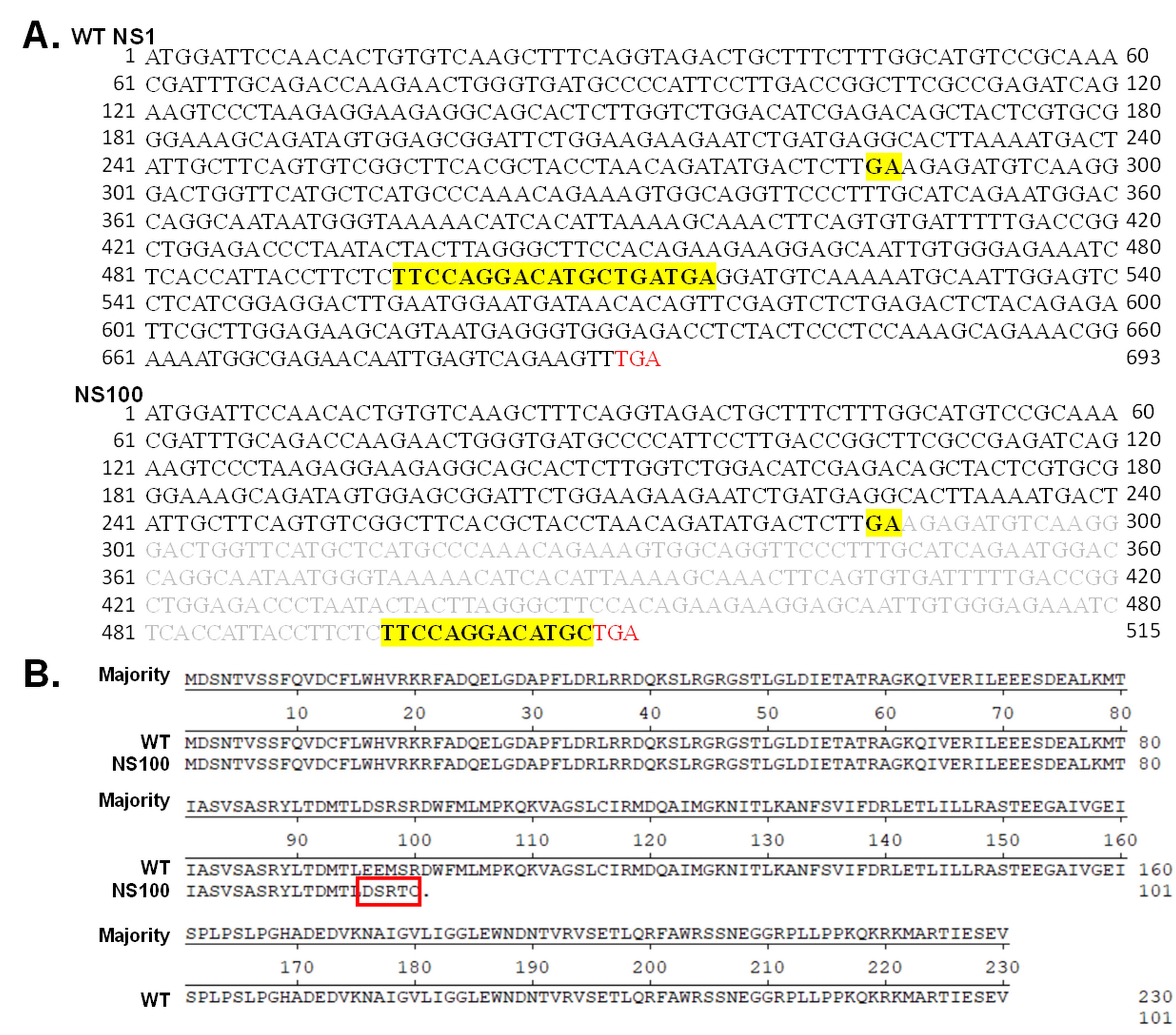

Supplement: Fig S1.tif [file TEMI_A_2556731_SM4356.tif]

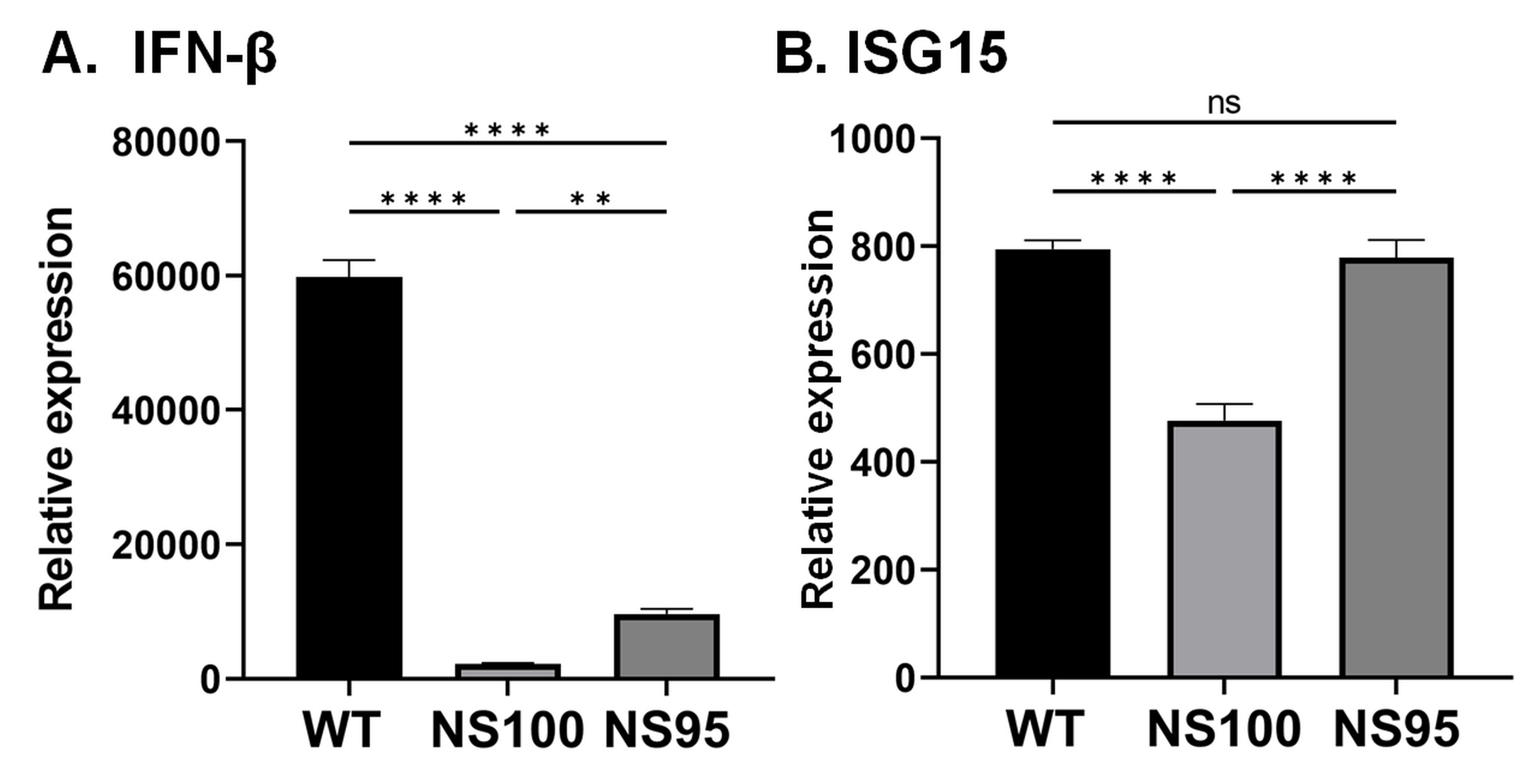

Supplement: Fig_S4.tif [file TEMI_A_2556731_SM4355.tif]

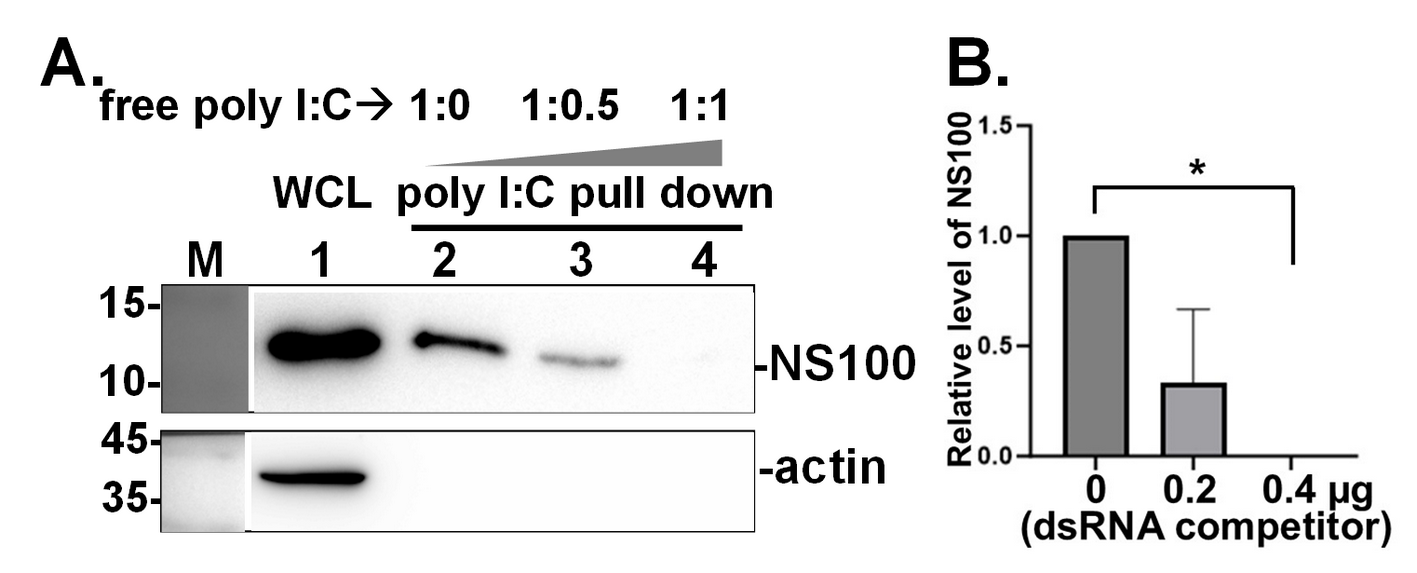

Supplement: Fig_S2.tif [file TEMI_A_2556731_SM4354.tif]
